# Oxygenation and function of endocrine bioartificial pancreatic tissue constructs under flow for preclinical optimization

**DOI:** 10.1177/20417314241284826

**Published:** 2025-01-23

**Authors:** Brenden N Moeun, Florent Lemaire, Alexandra M Smink, Hamid Ebrahimi Orimi, Richard L Leask, Paul de Vos, Corinne A Hoesli

**Affiliations:** 1 Department of Chemical Engineering, McGill University, Montreal, QC, Canada; 2 Department of Pathology and Medical Biology, University Medical Center Groningen, University of Groningen, Groningen, The Netherlands; 3 Department of Biological and Biomedical Engineering, McGill University, Montreal, QC, Canada

**Keywords:** Bioreactor, perfusion, diabetes, oxygenation, vascularization

## Abstract

Islet transplantation and more recently stem cell-derived islets were shown to successfully re-establish glycemic control in people with type 1 diabetes under immunosuppression. These results were achieved through intraportal infusion which leads to early graft losses and limits the capacity to contain and retrieve implanted cells in case of adverse events. Extra-hepatic sites and encapsulation devices have been developed to address these challenges and potentially create an immunoprotective or immune-privileged environment. Many strategies have achieved reversal of hyperglycemia in diabetic rodents. So far, the results have been less promising when transitioning to humans and larger animal models due to challenges in oxygenation and insulin delivery. We propose a versatile in vitro perfusion system to culture and experimentally study the function of centimeter-scale tissues and devices for insulin-secreting cell delivery. The system accommodates various tissue geometries, experimental readouts, and oxygenation tensions reflective of potential transplantation sites. We highlight the system’s applications by using case studies to explore three prominent bioartificial endocrine pancreas (BAP) configurations: (I) with internal flow, (II) with internal flow and microvascularized, and (III) without internal flow. Oxygen concentration profiles modeled computationally were analogous to viability gradients observed experimentally through live/dead endpoint measurements and in case I, time-lapse fluorescence imaging was used to monitor the viability of GFP-expressing cells in real time. Intervascular BAPs were cultured under flow for up to 3 days and BAPs without internal flow for up to 7 days, showing glucose-responsive insulin secretion quantified through at-line non-disruptive sampling. This system can complement other preclinical platforms to de-risk and optimize BAPs and other artificial tissue designs prior to clinical studies.

## Introduction

It is estimated that around 10 million individuals worldwide are living with type 1 diabetes (T1D), with prevalence expected to double by 2040.^1^ Although lifesaving, insulin therapy cannot regulate glucose levels as effectively as pancreatic islets.^2,3^ Consequently, patients with T1D carry additional healthcare costs, experience impacts on their quality of life, and can develop diabetic complications such as angiopathy, nephropathy, and neuropathy.^4–6^ Since the 1990s, islet allotransplantation has been shown to be an effective cell therapy for restoring glycemic control and improving the quality of life for patients with T1D.^7^ With advances toward insulin-producing cell sources from pluripotent stem cells^8–10^ or xenogeneic donors,^11^ the main remaining hurdles toward widespread application of cellular therapy for type 1 diabetes are ensuring long-term graft safety and efficacy.

As an alternative to hepatic islet transplants, devices, or scaffolds that enable graft containment, retrieval, immunomodulation,^12,13^ or protection from components of the immune system are being developed.^12,13^ Many of these strategies rely on the immobilization of islets in hydrogels or scaffolds that are not immediately vascularized or are inhibitive to vascularization. In these devices, the transport mechanisms for oxygen, other nutrients, and insulin are limited to diffusion. Passive mass transport in vivo can be sufficient for thin implants in small animal models,^14,15^ however in human trials, oxygen transport limitations, which can be exacerbated by foreign body responses, have led to hypoxia-mediated cell losses^16^ and the absence of vascular integration has resulted in subtherapeutic insulin responses despite the recovery of viable cells after explanation.^17^

Typical strategies to accelerate molecular transport include: (I; with internal flow) driving active blood or other fluid flow by connection to existing blood vessels or flow systems, (II, microvascularized) designs that rely on vascular ingrowth pre- or post-islet transplantation and (III, without internal flow) designs relying on surface vascularization or oxygenation. The first case relies on driving bulk fluid motion by creating a pressure gradient across pre-established flow channels within the device—for example by anastomosis to vasculature^18–20^ or via a pump.^21^ The second design typically relies on polymers with interconnected pores which allow or drive microvessel ingrowth. Islets may be introduced into these scaffolds at the time of implantation^22^ or into a compartment once vascularization has been established.^23–26^ The third design relies on surface vascularization or oxygenation of cell-impermeable devices such as microbeads, fibers, sheets, or pouches, where the surface area to volume ratio becomes a critical factor for islet survival and function.^27^

To rationally design encapsulation systems or extra-hepatic scaffolds for diabetes cellular therapy, it is important to understand how concentration gradients of nutrients such as oxygen, insulin and waste products will impact graft survival and function. Understanding the conditions that promote and limit these characteristics in human-scale tissues in the long-term is crucial when determining BAP specifications such as size, oxygenation strategy, and transplantation site. It is also well known that islet culture under flow (e.g. microfluidics) significantly improves pancreatic islet quality and better replicates in vivo conditions when compared to static cultures.^28,29^ However, most in vitro platforms for culturing artificial tissues focus on fundamental research, being designed for mm-scale tissues and studying sub-therapeutic cell densities. Perfusion systems such as packed bed columns or hollow fiber systems have been described by our group^30^ and others to study hydrogel-immobilized islets.^31–33^These bioreactors are typically designed for specific tissue geometries and provide limited capacity to monitor tissue viability in real-time.

We recognize that a complex bioartificial pancreas (BAP) will require more sophisticated culture systems to sustain proper oxygenation and function. It has been shown that perfusion culture utilizing microfluidics offers significant improvements in pancreatic islet quality and consistency in vitro when compared to static cultures without perfusion.^34^ However, as this system was not a closed loop, they were only able to culture cells for a limited period of 7 days due to the use of a low flow rate (25 µL/h). For larger structures like organs, bioreactors can be employed for culture before transplantation. While microfluidics excels in controlling parameters like flow rate and shear stress, such control is more challenging in bioreactors.^31^ Furthermore, a critical parameter for assessing performance is the ability to monitor the viability and function of the graft in real-time without the need for physical contact or disturbance of the specimen. Unfortunately, this capability is often lacking in current perfusion reactors.

Considering these challenges, we describe a novel customizable perfusion system capable of culturing tissue constructs for multiple days and assessing the dynamic function of bioartificial organs in vitro while applying different oxygen tension levels at tissue boundaries. We also demonstrate the option to monitor the viability of constructs containing fluorescently labeled cells in real time. We show that this platform can be applied to study beta cell survival and function for prominent endocrine BAP designs of vastly different geometry and composition.

## Materials and methods

### Perfusion system design and fabrication

#### Flow device

**Figure 1.**
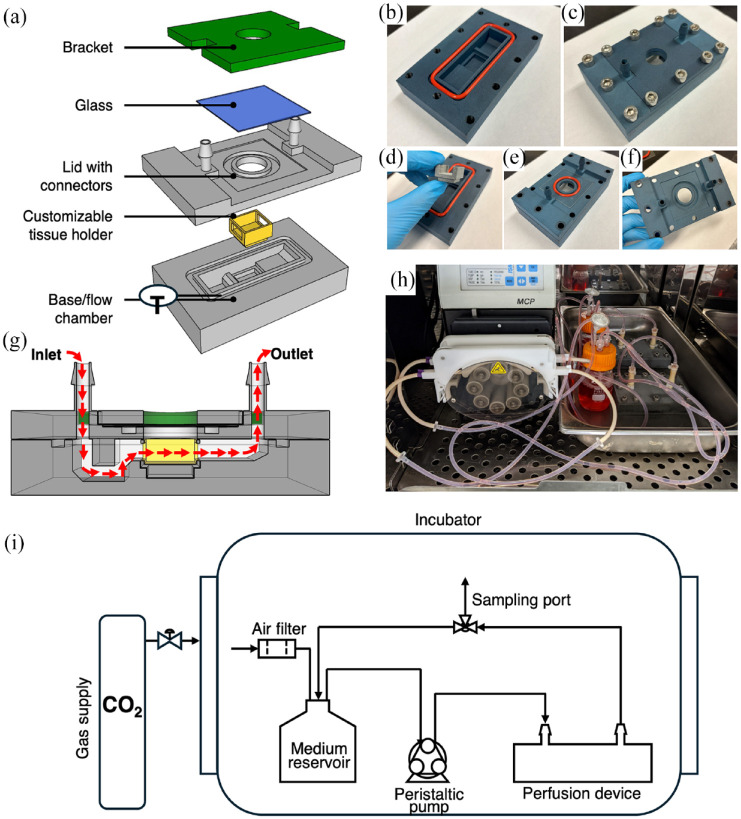
Flow device and perfusion system specifications: (a) General concept of flow device assembly. The base of the device holds a detachable tissue holder and a lid with integrated connectors encloses the flow chamber. A piece of glass covers the above-view imaging window and is kept in place with a bracket. (b) Cross-section of the flow device with red arrows indicating the general direction of flow. (c–g) Flow device parts and assembly after 3D printing. (h) Two assembled flow loops running inside of incubator. (i) Process flow diagram of the perfusion system. The flow device is connected to a vented reservoir, sampling port, and peristaltic pump via tubing and the system is placed within a cell incubator.

A flow device was designed using SketchUp software (Trimble) and 3D printed in a polyamide ink (PA12, Shapeways, Livonia, USA) using a multi jet fusion 3D printing service (shapeways.com). A customizable tissue holder that can detach from the device was designed and 3D printed from the same material to fit in the middle of the internal flow chamber, demarcating the tissue compartment (Figure 1(b) and (d)). The flow chamber’s base has a U-shaped inlet reservoir to facilitate even flow within the chamber and prevent bubbles from advancing into the tissue construct compartment.^35^ The lid and base of the device are sealed with an O-ring (McMaster-Carr, #231 Ø1/8, Aurora, USA) that fit into a groove that runs around the perimeter of the internal flow chamber (Figure 1(b)). Integrated barbed flow ports (ØID3/16) on the lid facilitate assembly and prevent leaking from the device inlet and outlet. An above-view imaging window is sealed with another O-ring (McMaster-Carr, #217 Ø1/8, Aurora, USA) sandwiched between a Corning^®^ Gorilla^®^ glass cover (McMaster-Carr, Aurora, USA) and the top of the device’s lid (Figure 1(e)). The glass is held down with a 3D printed bracket. The bracket, lid, and base were threaded (M6, 1.0 mm thread, 25 mm long, McMaster-Carr, Aurora, USA) after printing and are held together using 12 sealing socket head screws (McMaster-Carr, Aurora, USA). The overall device is 120.3 mm in length, 73.1 mm in width, and 105.7 mm in height (Supplemental Figure S1).

**Figure 2.**
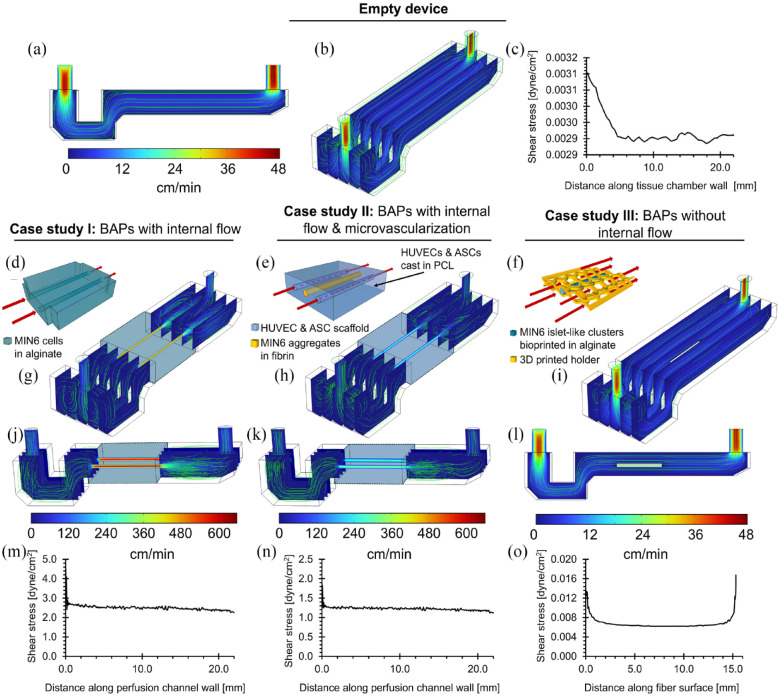
Computational flow dynamics (CFD) modeling of flow device chamber and case studies. (a–c) Empty flow chamber: (a) Longitudinal cross-section. (b) Angled view. (c) Shear stress profile on tissue holder walls. (d–f) Schematics of case studies: (d) BAP with internal flow (inlet flow rate was 8 mL/min); (e) BAP with internal flow and microvascularization (inlet flow rate was 4 mL/min); (f) BAP without internal flow (inlet flow rate was 4 mL/min). (g–i) Angled view of CFD models for each case study respectively. (j and l) Longitudinal cross section of CFD models for each case study respectively. (m–o) Shear stress profile for each case study respectively. For all CFD models, streamlines are shown in green.

For experiments with bioprinted fibers, the construct was suspended within the tissue holder using a stack of custom-made grid layers that allowed flow above and below the construct (Figure 2(f)). The grid layers were designed using SolidWorks software and 3D printed with polylactic acid using a fuse deposition modeling platform (Ender 3, Shenzhen, China). The stereolithography files used to 3D print all parts are provided in the Supplemental Material.

#### Flow loop

The flow device was connected to the perfusion loop using tubing reducers (Ø3/16 to Ø3/32, McMaster-Carr, Aurora, USA) and short pieces of tubing (Ø3/16, MasterFlex, Cole Parmer, Vernon Hills, USA). The loop included a 100-mL medium reservoir that connected to the inlet and outlet of the flow device. A vent with a 0.2-μm filter (Fisher Scientific, Pittsburgh, USA) allowed for airflow and gas exchange. A sampling port was created in the loop using an at-line tee reducer (McMaster-Carr, Aurora, USA). Pump tubing (Ø1/10 for case studies I and III, Ø1/32 for case study II, Ismatec, Wertheim, Germany) was used to connect the loop to a peristaltic pump (MCP Standard, Ismatec). The entire perfusion system (flow device, loop, reservoir, and pump) was placed within a 37°C, 5% CO_2_ incubator during all flow experiments. For studies where the oxygen tension was set below atmospheric levels, the flow system was placed within a hypoxia incubator (Heraeus, HERAcell 150). Figure 1(h) illustrates the entire perfusion system setup as a simplified process flow diagram. All components including device materials are autoclavable. The flow loop, aside from the chamber components needed for tissue insertion, were assembled prior to autoclaving.

### Computational fluid dynamics

A stereolithography file of the internal flow chamber was imported directly into COMSOL Multiphysics 5.5 software. Cell culture medium flow (μ = 0.69 mPa s; ρ = 0.993 g/cm^3^) was modeled with a no-slip condition for all internal walls in the chamber. The laminar solver with a relative tolerance of 0.001 was implemented. Mesh independence was determined with velocity profiles. Various geometries representative of experimental conditions were modeled including an empty flow chamber, a tissue construct with 2 perfusable channels (case studies I and II), and a fiber suspended within the tissue holder (case study III). We assumed incompressible, Newtonian fluid, constant viscosity, steady 1D plug flow at the inlet (8 mL/min for case study I and 4 mL/min for case studies II and III), and rigid, traction free walls.

### Cell culture

Mouse insulinoma 6 (MIN6) cells were generously gifted from Dr. Jun-ichi Miyazaki (Osaka University) and were grown in adherent polystyrene T-flasks treated for tissue culture (Sarstedt, Numbrecht, Germany) at 37°C and 5% CO_2_.^36^ The cells were cultured in Dulbecco’s modified Eagle’s medium (DMEM, Fisher Scientific, Grand Island, USA) supplemented with 10% fetal bovine serum (FBS; Fisher Scientific, South Logan, USA), 100 U/mL penicillin/streptomycin (Fisher Scientific, Grand Island, USA), 2 mM L-glutamine (Fisher Scientific, Grand Island, USA), and 50 μM β-mercaptoethanol (MilliporeSigma, St. Louis, USA). Medium was changed every 48 h until cells reached ~90% confluency. Cells were then passaged or harvested using TrypLE Express Enzyme (Fisher Scientific, Grand Island, USA). Passage numbers were kept below 50 (received at p17, created master from p19, and working banks from p27) and we used cell suspensions with a viability of at least 85%. Cell suspension viability was measured using trypan blue (MilliporeSigma, St. Louis, USA).

To generate islet-like clusters, MIN6 cells were aggregated using Aggrewell plates (Stem Cell, Vancouver, BC, Canada) according to manufacturer’s instructions at a seeding density of 500 cells per microwell. MIN6 clusters were harvested from Aggrewell plates after 48 h and passed through a 40 µm cell strainer to remove unattached free cells. Clusters were cultured in the same medium used for single MIN6 cells.

The use of human islets in this study was approved by the McGill Research Ethics Board. Human islet isolation was performed by the McGill University Health Centre Human Islet Transplant Laboratory (Montréal, QC, Canada). Islets were cultured in CMRL medium (Fischer Scientific, Grand Island, USA) supplemented with 2% human albumin serum (MillliporeSigma, St. Louis, USA) and 100 U/mL penicillin/streptomycin. These human islets have been used for research as they did not reach therapeutic grade for transplantation in human.

Human umbilical vein endothelial cells (HUVECs; Lonza, passages 4–6) were provided by the Endothelial Cell Facility of the UMCG. HUVECs were cultured in endothelial cell growth medium-2 (EGM-2, Lonza, Allendale, USA) supplemented with microvascular EGM-2 SingleQuot Kit Supplements and Growth Factors (Lonza). Human adipose-derived stromal cells (ASCs, passages 4–6) were isolated as previously described by Navarro Chica *et al.* (Islets 2022) from living kidney donors at the UMCG. These samples were anonymously donated with informed consent after approval from the UMCG ethical review board. ASCs were cultured in DMEM 4.5 g/L D-glucose supplemented with 50 U/mL penicillin, 50 mg/mL streptomycin, 2 mM L-glutamine, and 10% heat-deactivated FBS (Thermo Fisher Scientific, Bleiswijk, The Netherlands).

### Fabrication methods of pancreatic constructs

BAPs with internal flow can be categorized into two different approaches. The first approach involves directly connecting the construct to blood vessels while the islets are encapsulated in a hydrogel. Although internal flow can be crucial for maintaining proper oxygenation and providing beta cells with direct access to glucose for detection and insulin secretion, studies on microencapsulated islets have shown that sufficient oxygen diffusion alone can maintain cell survival and function over time to potentially reverse diabetes. In this context, interactions between islets and endothelial cells are not always necessary. Such constructs are represented by our Case Study I. The second approach focuses on using vascularized encapsulation constructs to enhance and accelerate graft integration into the blood circulation post-transplantation. This approach is exemplified by our Case Study II. In this study, we aimed to evaluate the feasibility of co-culturing endothelial cells with islets.

#### Case study I: with internal flow

To prepare perfusable pancreatic tissue constructs, MIN6 cells were mixed with a 2.5% w/v alginate solution (IFF Nutrition & Biosciences through DuPont, Protanal LF 10/60) containing 30 mM calcium carbonate (CaCO_3_, VWR, Center Valley, USA) to result in a 40 × 10^6^ cells/mL cell density. Glucono-δ-lactone (MilliporeSigma, St. Louis, USA) dissolved in HEPES buffered saline (Fisher Scientific, Fair Lawn, USA) was then added to a final concentration of 60 mM. All solutions were prepared with a pH of 7.4. Alginate and CaCO_3_ solutions were sterilized by autoclaving at 121°C for 30 min whereas the glucono-δ-lactone was filter-sterilized using a 0.2-μm filter immediately before use. The cell-laden alginate mixture was then cast directly into the tissue holder, around two needles (Fisher Scientific, Whitby, Canada) that were suspended within the holder. The outer walls of the tissue holder were wrapped with a single layer of tape prior to autoclaving to prevent outflow and to keep the needles in place. The mixture was then left to gel at room temperature for 30 min. Following, the needles were pulled out to create hollow channels and the tape was removed before placing the loaded tissue holder within the perfusion device.

The resulting tissues were then cultured in the perfusion system at a flow rate of 4 mL/min/channel with 80-mL of complete DMEM in the reservoir at 37°C, 5% CO_2_, and atmospheric oxygen tensions for 2 days. The flow device used in these experiments were modified as to exclude the imaging window for experimental simplicity. Complete medium used for perfusion was supplemented with an additional 5 mM of calcium chloride (MilliporeSigma, St. Louis, USA) to prevent de-gelling.

#### Case study II: with internal flow and microvascularized

Porous polycaprolactone (PCL; average Mn 80,000; Sigma-Aldrich, Zwijndrecht, The Netherlands) scaffolds were fabricated as previously described.^37^ In these 10 mm × 15 mm scaffolds, three channels were created by using a 23 G needle (Becton Dickinson, Franklin Lakes, USA). The two outer channels ensured media flow in the flow chamber and were, therefore, created over the full length of the scaffold. The middle channel was used for placing insulin-producing cells, to this end, this channel was created over 5/6 of the length of the scaffold to prevent outflow of these cells. During the HUVEC and ASC seeding process described below, these channels were kept patent by placing polyethylene tubing (0.5 mm × 1 mm; Brinkman’s-Gravenzande, The Netherlands). Before use, these scaffolds were sterilized by an overnight incubation in 70% ethanol.

To support the insulin-producing cells, endothelial cells and ASCs were incorporated into the PCL scaffolds. A 90:10 mixture of these cells was suspended in a rat tail collagen type 1 (Gibco; Life Technologies, Bleiswijk, The Netherlands) solution, with a final concentration of 2 million HUVECs and 0.2 million ASCs per scaffold. The scaffolds were incubated within the cell solution for 25 min on ice while shaking and periodic (every 5 min) manual mixing to avoid sedimentation, after which they were placed for 30 min in a 37°C incubator to allow collagen gelation. After collection, approximately 2500 MIN6 aggregates were mixed with 10 μL rat fibrinogen (2 mg/mL; Stago, Leiden, The Netherlands). Before adding 0.1 μL rat thrombin IIa (100 U/mL; Stago), to this mixture of fibrinogen and MIN6 aggregates, the tubing from the middle scaffold channel was removed. This allowed the injection of the fibrin/MIN6 mixture into the channel. Scaffolds were incubated for 15 min at room temperature and subsequently 15 min in a 37°C incubator to ensure the fibrin formed a gel. The scaffolds were then placed into pre-warmed flow chamber medium that consisted of 90% EGM2-MV medium and 10% MIN6 culture medium.

Before placing the scaffolds in the flow chamber, the outer tubing was removed. Two scaffolds containing the three cell types, as described above, were placed in the tissue holder of the flow chamber. The dead end of the middle channel was facing the flow chamber outlet to ensure MIN6 aggregates didn’t flow out of the scaffold during the experiment. After assembly, the flow chamber was placed in a 37 °C incubator (21% O_2_ and 5% CO_2_) and connected to the pump to start the flow (4 mL/min). A third scaffold was placed in a 10-cm Petri dish with flow chamber medium, as static control. The constructs were cultured for 3 days.

#### Case Study III: without internal flow

MIN6 clusters or human islets were encapsulated in alginate core-shell fibers using Aspect Biosystems 3D (Vancouver, BC, Canada) bioprinter (RX1) placed in a biosafety cabinet. For the core of the fibers, 21,000 MIN6 clusters or 15,000 human islets were mixed in a final 1.5% alginate solution with DMEM medium (resulting in a 21,000 MIN6 clusters/200 µL and 15,000 human islets/200 µL cell density respectively). For the shell of the fibers, acellular alginate was prepared at final 2% concentration. Each solution was loaded in the bioprinter according to manufacturer’s instructions. Alginate was externally gelled by printing fibers directly in a 10 mL calcium chloride and barium chloride solution (HEPES 10 mM, 100 mM calcium chloride, 2 mM barium chloride). After printing, fibers were washed for 3 min in medium and placed incomplete DMEM medium (37°C, 5% CO_2_). For each independent experiment, one unique long fiber was printed and cut in different pieces (16 mm length, 1.2 mm diameter, 18 mm^3^ volume, and around 300 MIN6 clusters or human islets per fiber) for culture either in static conditions or in the perfusion device. Four fibers inserted in a 3D printed holder and placed in the tissue holder of the perfusion device. The flow loop and device were then filled with 40 mL of completed DMEM. MIN6 clusters encapsulated in fibers were cultured for 7 days and encapsulated human islets were cultured for 2 days at a 4 mL/min flow rate. Human islets-containing fibers were cultured in two distinct perfusion devices. One was placed in an atmospheric incubator (21% O_2_, 5% CO_2_), while the other was placed in an oxygen-controlled incubator (5% O_2_, 5% CO_2_).

### Glucose-stimulated insulin secretion tests

After perfusion culture, we tested the dynamic glucose-stimulated insulin secretion (GSIS) function of the tissue construct. The loop was first flushed of cell culture medium and configured into a single-pass format where instead of cycling the perfusate, the liquid leaving the device would be captured in sampling tubes. There was a synchronization step where the tissue was perfused with low-glucose (2.8 mM, MilliporeSigma, St. Louis, USA) Kreb’s buffer for at least 2 h at 4 mL/min/channel. The base Kreb’s buffer used for these experiments was prepared by dissolving 130 mM sodium chloride (NaCl, MilliporeSigma, St. Louis, USA), 4.7 mM potassium chloride (KCl, 20 mM, MilliporeSigma, St. Louis, USA), 1.2 mM magnesium sulfate (MgSO_4_, MilliporeSigma, St. Louis, USA), 10 mM HEPES, 2.5 mM Calcium chloride dihydrate (CaCl_2_ · 2H_2_O, MilliporeSigma, St. Louis, USA), 5.0 mM sodium bicarbonate (NaHCO_3_, MilliporeSigma, St. Louis, USA) and 0.5% w/v bovine serum albumin (BSA, MilliporeSigma, St. Louis, USA) in reverse osmosis water. For case study II, sodium bicarbonate was omitted and 25 mM HEPES was used instead. The pH was then brought to 7.4 using 2 N sodium hydroxide (Fisher Scientific, Whitby, Canada).

Following the synchronization stage, the GSIS function of BAPs were evaluated in four stages: (1) a basal stage in low-glucose Kreb’s; (2) a stimulation stage in high glucose Kreb’s (16.7 mM glucose); (3) a second basal stage at low glucose; (4) a decoupling stage in KCl Kreb’s Krebs buffer (20 mM) Meanwhile, samples were collected periodically at the outlet. The sampling frequency and stage durations for the three case studies evaluated in this work are summarized in Table 1. At the endpoint case study III (7 days for MIN6 clusters and 2 days for human islets), alginate fibers were de-gelled using 0.1 mM EDTA solution (Fischer Scientific, Whitby, Canada).

**Table 1.** GSIS parameters for three case studies.

Parameter	Case study I: BAP with internal flow	Case study II: internal flow w/vascularization	Case study III: BAP without internal flow	
			MIN6 clusters	Human islets
First basal time (min)	30	40	30	30
Stimulation time (min)	60	40	40	30
Second basal time (min)	60	40	60	60
Depolarization time (min)	30	–	30	10
Collection frequency (min)	5	5	2	5

All collected samples were centrifuged at 1200 rpm for 5 min to remove any possible debris. The supernatant was then collected and stored frozen at −20°C until analysis. Islets were washed, centrifuged, and resuspended in acid/alcohol solution for total insulin content extraction. The insulin content of the samples was measured using a mouse insulin ELISA kit (case studies I and III: Cedarlane, 80-INSMS-E01; case study II: Mercodia, Uppsala, Sweden) according to the manufacturer’s instructions. The optical absorbance was measured using a Benchmark Plus Microplate Spectrophotometer (Bio-Rad) for case studies I and II and a BioTek Epoch 2 (BioSPX, Abcoude, The Netherlands) for case study II.

### Cell viability

#### End-point viability

To assess the viability of cells within the constructs with internal flow, fluorescent live/dead staining was performed on cross sections of the tissues. Of note, most flow experiments were limited to 2–3 days as to minimize the effect of proliferation on the OCR of BAPs. Thin cross-sections (~1 mm thickness) were sliced manually using a razor blade and then incubated in a HEPES-buffered staining solution containing 5 mM calcium chloride, 10% v/v DMEM, 4 μM calcein AM (Thermo Fisher Scientific, Bedford, USA), and 30 μM propidium iodide (Thermo Fisher Scientific, Eugene, USA) for 30 min at room temperature. The slices were then rinsed with HEPES buffered saline solution and imaged using an Olympus IX81 inverted microscope system and MetaMorph acquisition software. The whole cross section was imaged by programming the microscope system to capture sequential pictures in a pre-defined grid pattern. The resulting images were then stitched together and processed using Fiji software. Viability of constructs without internal flow (case study III) was evaluated at the endpoint of the study using calcein and propidium iodide staining. The viability of the three cell types within the scaffold constructs (case study II) was also determined using live/dead staining. Briefly, the scaffolds were removed from the chamber or the static culture and cut into two halves. One scaffold half was incubated for 30 min at room temperature in 1 uM calcein AM and 1 uM ethidium bromide (live/dead staining kit Molecular Probes; Life Technologies). After several washes, the scaffolds were imaged using an EVOS^®^ FL Cell Imaging System (Life Technologies).

#### Real-time viability

Tissue constructs with 40 × 10^6^ cells/mL and a central single channel were cast into the tissue holder as described above. The MIN6 cells used in these experiments were generously gifted from Dr. James Johnson and Dr. Yu Hsuan Carol Yang and constitutively express green fluorescent protein (GFP) via a Förster resonance energy transfer (FRET) mechanism (MIN6 FRET).^38^ A second layer of acellular alginate was then cast on top of the tissue layer to add depth. The layers were 3.2 and 3.9 mm thick, respectively. A glass cover slip was then placed directly on top of the acellular layer to prevent oxygen diffusion from above and the tissue construct was loaded into the flow device. The flow device was placed in an IVIS Lumina III imaging system (Perkin Elmer) with the temperature set to 37°C. The flow loop ran from the flow device to the medium reservoir and pump which were held in a separate incubator set at 37°C and 5% CO_2_. Fluorescent images were taken every 30 min for 48 h (excitation: 500, emission: 583). Only signal coming out of the imaging window was considered in the region of interest when measuring the radiance and radiance efficiency. Images and signal were processed using Living Image software (Perkin Elmer). Following the real-time imaging and perfusion culture, constructs were sliced, and live/dead imaging was performed as described above except without calcein AM.

### Metabolic sampling and analysis

The metabolic activity of our BAPs with internal flow was evaluated through periodic perfusate samples from the perfusion loop for case study I and III. Throughout the 2-day culture for case study I, 0.5-mL medium samples were extracted from the loop via the sampling port. For case study III, 0.5 mL medium samples were extracted from the loop after 1, 3, and 5 days. Samples were kept frozen at −20°C until analysis. Metabolic analysis for glucose, glutamine, and lactate were performed using the Cedex Bio Analyzer (Roche) and the corresponding test kits according to the manufacturer’s instructions.

### Oxygen transport modeling

Tissue constructs with either 40 × 10^6^ or 10 × 10^6^ cells/mL and two perfusion channels were prepared as described above and perfused for 2 days at 4 mL/min/channel, 37°C, and 5% CO_2_ (flow device without an imaging window). Tissue cross sections were stained as described above and dimensions of viable cell regions were measured using Fiji software (version 1.9.0).^39^ Theoretical oxygen profiles were generated using COMSOL Multiphysics 5.5 software (fine mesh size) as previously described in a previous publication.^40^ Briefly, we assumed only radial oxygen transport, a constant oxygen tension at the channel walls, and no diffusion at the outer edges of the tissue slice to solve the diffusion model described in equation (1).

(1) $$-D_{eff}\nabla^{2}C_{O_{2}}=R$$

Where $D_{eff}$ represents the effective diffusivity of oxygen through the beta cell-laden alginate, $C_{O_{2}}$ represents the oxygen tension as a function of the radius, and R represents the oxygen consumption rate. As described in equation (2), we modeled the oxygen diffusivity as a weighted average of the diffusivity of oxygen through alginate ( $D_{alg}$ ) and the diffusivity of oxygen through cells ( $D_{cells}$ ).

(2) $$D_{eff}=D_{cells}X+D_{alg}(1-X)$$

Further, to define R , we use the Monod oxygen consumption kinetics described in equation (3).

(3) $$R=\left[\frac{-R_{O_{2}}\cdot C_{O_{2}}}{C_{O_{2}}+K_{s}}\cdot \delta \left(C_{O_{2}}>C_{cr}\right)\right]\cdot X$$

Where $R_{O_{2}}$ represents the maximum oxygen consumption rate, *K_s_* represents the Monod coefficient for oxygen consumption, δ is the Heaviside function, $C_{cr}$ represents the critical oxygen tension below which cell necrosis occur; and X represents the volumetric cell fraction. All model parameters are summarized in Table 2.

**Table 2.** Oxygen transport modeling parameters.

Parameter	Value	Source
MIN6 Maximum oxygen consumption rate (OCR, $R_{O_{2},M6}$ )	0.129 mol/s/m^3^	Avgoustiniatos et al.,^41^ Buchwald,^42,43^ and Coronel et al.^44^
Islet Maximum oxygen consumption rate (OCR, $R_{O_{2},HI}$ )	0.034 mol/s/m^3^	Buchwald^42^
MIN6 Monod constant for oxygen consumption ( $K_{s,M6}$ )	0.62 μM	Buchwald,^42^ Johnson et al.,^45^ and Avgoustiniatos and Colton^46^
Islet Monod constant for oxygen consumption ( $K_{s,HI}$ )	1 μM	Buchwald^42^
Necrotic oxygen tension ( $C_{cr}$ )	0.1 mmHg	Johnson et al.,^45^ Suszynski et al.,^47^ and Cao et al.^48^
Oxygen diffusivity in alginate ( $D_{alg}$ )	2.8 × 10^−5^ cm^2^/s	Mehmetoglu et al.^49^ and Cristea et al.^50^
Oxygen diffusivity in cells ( $D_{cells}$ )	1.24 × 10^−5^ cm^2^/s	Buchwald^42^ and Johnson et al.^45^
Atmospheric oxygen tension	140 mmHg	Martinez et al.^51^
Venous oxygen tension	40 mmHg	Ortiz-Prado et al.^52^

### Histology

For case study II, half of the scaffold unused during live/dead staining was placed in 2% paraformaldehyde overnight, after which it was processed for paraffin embedding. Sections of 4 µm were used for hematoxylin and eosin staining of the scaffolds. Images were obtained using the Leica DM 2000 LED microscope with a Leica DFC450 camera (Leica Microsystems, Amsterdam, The Netherlands).

### Statistical analysis

GraphPad Prism software (version 9.5.0, Boston, USA) was used to perform the statistical analysis. Data sets were first tested for normality using the Shapiro-Wilk test. One-way ANOVA and Tukey post-hoc testing were used to determine time effects while a series of unpaired t-tests were conducted to compare different conditions. The standard error of the mean (SEM) for line plots are presented as shaded areas. Reported values and bar graph data represent mean ± SEM. All experiments were conducted in triplicate (*n* = 3) unless otherwise stated. Statistical significance is reported using the following asterisk notation: *p < 0.05, ** *p* < 0.01, and *** *p* < 0.001.

## Results

### Perfusion system design and fabrication

Three BAP configurations were considered as case studies when developing and testing the perfusion system.

#### With internal flow (or directly perfusable)

Examples of this scenario include devices implanted via anastomosis to existing blood vessels^53,54^ and those that employ an external oxygenated fluid pump.^17^ Here, single channels were studied as simplified base units for these devices as these data can be scaled up to represent more complex perfusable networks. We fabricated these devices using alginate, a commonly used material for islet encapsulation, due to its biocompatible and immunoprotective properties.^13^

#### With internal flow and microvascularized

Another prevalent BAP oxygenation strategy is to rely on angiogenesis, neovascularization, and vascular ingrowth following implantation. Devices that contain blood vessel-forming cells such as endothelial cells^55^ and/or permit new blood vessels to grow directly into the device post-transplantation^56,57^ are included within this case study. In this work, we model these BAPs with a perfusable and porous scaffold laden with endothelial cells and ASCs. The scaffold is made of polycaprolactone, a material known for its biocompatibility.^58^ We included two internal flow channels to model a situation where temporary flow (e.g. via vascular anastomosis or a mechanical pump) would be provided internally until microvasculature is established.

#### Without internal flow

In BAPs without internal flow oxygen and insulin transport rely on superficial diffusion rather than bulk fluid motion. As such, this case study encompasses pouches,^59,60^ sheets,^61,62^ fibers, and other configurations without internal vascularization. We model this classification of devices using a single bioprinted fiber suspended within a flow field. The flow field simulates a transplantation site by maintaining a constant oxygen tension during experiments and we selected a bioprinted fiber as a base unit since 3D printed filaments can be used to generate various macro-geometries. Therefore, for this case study, we explored the geometric configuration with a minimal oxygenation limitation.

To accommodate these various tissue geometries, the perfusion system was built based on the design criteria described in Table 3 to create a flexible platform compatible with various tissue geometries, with or without internal flow. The holder for the tissue constructs is detachable and 3D printed to allow the user to modify only the single piece to suit their geometric and experimental (e.g. flow around or flow through the tissue) parameters.

**Table 3.** Design criteria for flow device.

Design feature	Design criterion	Comments
Customizable setup	Devices with or without internal flow	Flow through or around a tissue can simulate devices with or without internal vascularization
	Flexible with tissue geometries	A removable tissue holder facilitates customization without recreating the entire flow device
	Adjustable gas composition	The flow system can be placed inside a gas-controlled incubator (alternatively, a gas exchange system could be used^30^)
	Adaptable to scale	3D printing allows tissue holder to be modified to fit smaller tissues and the rest of the flow device to be scaled up or down
Cell culture considerations	Facilitates aseptic assembly	The design minimizes the number of parts and preassembled components can be autoclaved to reduce risks of contamination
	Watertight	O-rings fitted to prevent leaks
	Easy to load tissues	Tissue holder is removable, and constructs can be loaded separate from the device body or made directly inside of it
	Can sterilize with mainstream methods	All materials can be autoclaved repeatedly with no observable warping and no damage to water-sealing
	Cytocompatible materials	Polyamide and polylactide show low cytotoxicity^63,64^
	Low dead volume	Reduce costs and measured response times in GSIS assays. Device dead volume: 55 mL if empty with tubing, ~50 mL with inserted tissues
Flow	Laminar, uniform, fully developed flow	Entry and exit chambers to distribute flow and avoid disturbance
	Adjustable flow speeds	Flow rate is variable (0.5–30 mL/min tested without leaks)
Accessibility	Use of standard sizes and fittings	Aside from the 3D printed parts, all other pieces are commercially available and the connectors on device are standard size
	Easy to manufacture and modify	3D print parts can be fabricated through a 3D printing service and CAD files are available in the supplementary files.
Experimental versatility	Imaging	Top of perfusion device has a glass window for real-time imaging of tissues with upright systems (e.g. small animal imager)
	Tissue retrievability	Tissues can be recovered for post-culture analyses and fixing
	Medium sampling	Sampling port at the outlet for periodic sampling while maintaining aseptic conditions

### Modeling flow through or around tissues

In addition to the oxygenation strategy in BAPs, the success of these devices can also depend on the oxygen tension of the transplantation site. Generating a uniform, laminar, and steady flow profile within the flow device facilitates the simulation of a transplantation site while also permitting the option for perfusion. Laminar flow is also important to consider when considering the mass transport to and from BAPs (e.g. insulin response). The internal physical features of the flow device to achieve these fluid mechanics in the absence of any tissue insert were simulated using computational fluid dynamics (CFD) modeling (Figure 2(a) and (b)). The inlet and outlet reservoirs were effective in preventing backward flow, leading to smooth streamlines throughout the chamber despite circulation in the reservoirs.

To reflect different tissue geometries, the fluid mechanics were modeled with constructs containing two (cases I and II) internal flow channels or for flow around a single suspended fiber (case III). For tissues with internal flow (cases I and II), a rapid increase in fluid flow rate was observed as fluid passed from the inlet reservoir and into the channels, as expected (Figure 2(g), (h), (j), and (k)). Due to this sudden change in speed, circulation occurs within the outlet reservoir before exiting the flow chamber, but the flow remains laminar (Re < 50). Suspending a single fiber within the tissue compartment (case III) does not substantially affect the flow dynamics within the chamber as the system remains laminar (Figure 2(i) and (l)). Overall, fully developed unidirectional laminar flow was observed within >95% of the length of the channels (cases I and II) or around suspended fibers (case III) in all three configurations, with velocities ranging between 0.5 and 78.5 mm/s and wall shear stresses ranging from 0.0029 to 4.5 dyne/cm^2^. According to literature, the reported shear stresses are not expected to be damaging to cells.^65^

### Case study I: Viability, metabolism, and function of BAPs with internal flow

#### Beta cell survival

**Figure 3.**
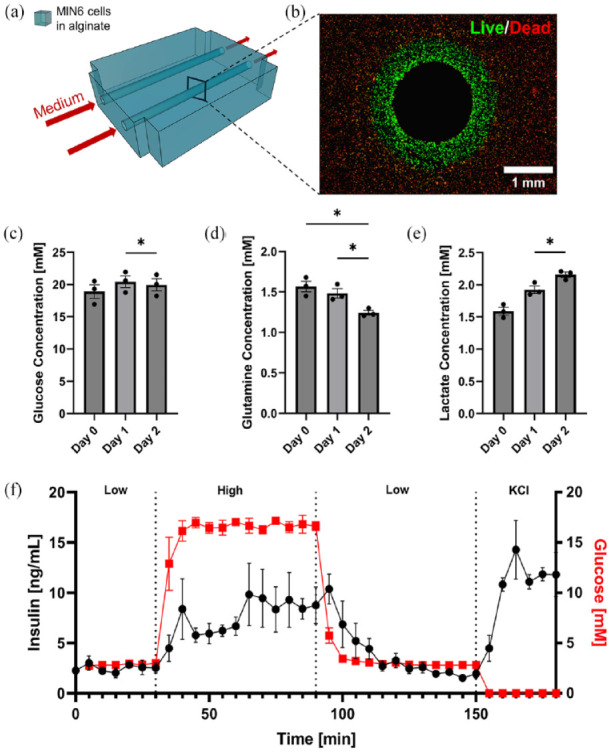
Viability and function of artificial pancreatic tissues with internal flow (40 × 10^6^ cells/mL starting density) after 2 days of perfusion culture: (a) Schematic of experimental setup: artificial pancreatic tissues are irrigated with two hollow channels and cultured under perfusion for 2 days. (b) Viability stain of a crosssection of one of the channels. Live cells are stained green (calcein-AM) and dead cells in red (propidium iodide). (c–e) Quantification of metabolites in perfusion medium samples that were periodically taken throughout the 2-day perfusion. Glucose, glutamine, and lactate concentrations are shown, respectively *p < 0.05. (f) Dynamic GSIS function of artificial pancreatic tissue after 2 days of perfusion culture (4 mL/min/channel). All error bars indicate the standard error of the mean (*n* = 3).

To establish proof-of-concept of cell survival and monitoring within the system, dispersed MIN6 cells were cultured for 2 days in the simplest configuration (case I), whereby the tissue consisted of a cell-laden alginate block with two internal flow channels. After the 2 days of perfusion culture, only cells within a distinct distance from the perfusion channels remained viable (Figure 3(b)). This defined a viability region that spanned 0.55 ± 0.02 mm radially and cells outside of this range were dead; herein referred to as the viability radius.

#### Beta cell metabolite monitoring

To determine whether the system would be appropriate to quantify nutrient consumption rates in real-time, medium samples were periodically collected from the outlet line. We measured glucose and glutamine, two key molecules for ATP production of islets, and already present in the culture medium, to assess beta cell consumption over time. We also assessed lactate level in medium samples, a molecule secreted in anaerobic conditions. Based on our analysis of metabolites between days 2 and 3 in the perfusate, cell metabolism appeared to rely oxidative phosphorylation based on the ~0.5 molar conversion ratio between lactate production and glucose consumption (Figure 3(c) and (d)). As expected for MIN6 cells, glutamine consumption was significant. While the rate of glucose consumption remained constant, a significant time effect was found in the decrease in glutamine (*p* = 0.009). This finding is consistent with the literature leading up to this study as glutamine has been described as a significant carbon and nitrogen source for MIN6 cells and its uptake has been linked to insulin response mechanisms as well as other cell functions.^66–68^ A significant time effect was also found in the increase in lactate (*p* = 0.008) related to the lack of oxygen for living cells. The presence of lactate in the fresh culture medium was unexpected but could be attributed to the addition of fetal bovine serum in the medium. Using the previously described viability radius, and assuming that the cell number did not change significantly (i.e. no significant cell expansion) between 24 and 48 h, the cell specific glucose and glutamine consumption rates can respectively be estimated to be 3.2 ± 0.001 and 1.3 ± 0.0002 fmol/min/cell which are values comparable to the literature.^69^ These results demonstrate the capacity for real-time, non-invasive monitoring of perfusion culture medium for metabolic balances and other functional analyses.

#### Beta cell function

To determine the dynamics of insulin release from devices with internal flow, step changes in glucose concentration were applied at the device inlet. The measured secreted insulin profile shows a 2-phase insulin release dynamic (Figure 3(f)), consistent with previous observations made with microencapsulated MIN6 cells studied under perifusion.^70^ Depolarization elicited a rapid spike in insulin secretion (2570 ± 200 ng total insulin). Overall, we observe a delay in insulin response by 10 min after switching to the high glucose condition. This delay accounts for the approximately 6 min required to clear the dead volume and ~3 min for insulin diffusion reported by others for non-encapsulated beta cells in perifusion.^71^ Constructs that were stained before and after conducting the GSIS assay showed comparable viability radii (data not shown).

#### Real-time viability

**Figure 4.**
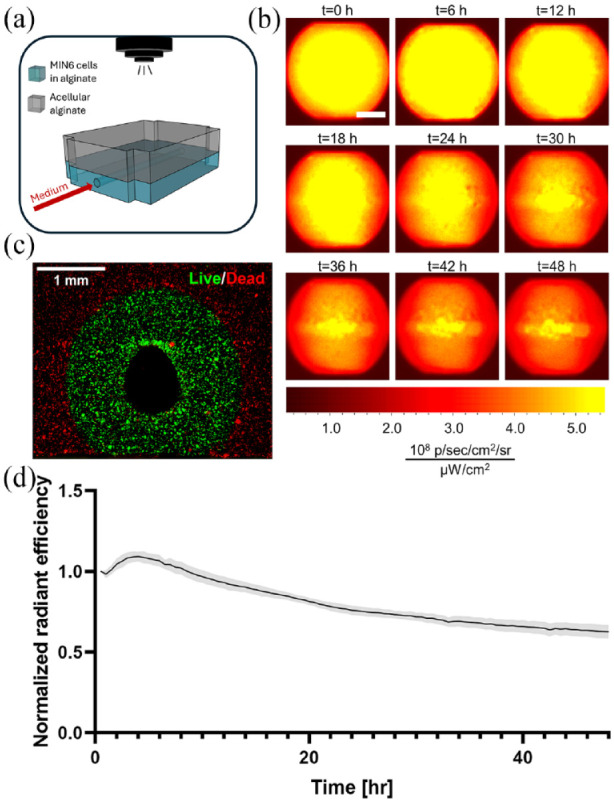
Real-time tracking of cell viability in pancreatic tissue constructs with internal flow (40 × 10^6^ cells/mL starting density): (a) Schematic of experimental setup. (b) Above-view fluorescent images of a single-channel pancreatic tissue construct monitored over 48 h (scale bar: 5 mm). (c) Viability of a cross-section of the tissue construct with green indicating live cells and red marking dead cells (propidium iodide). (d) Quantification of fluorescent signal from the tissue construct over 48 h. The shaded area represents the standard error of the mean, *n* = 3.

**Figure 5.**
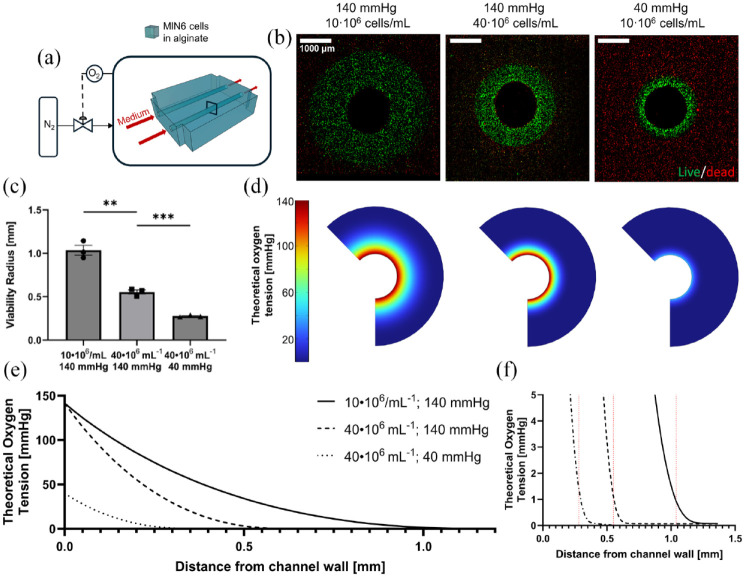
Modeling the viability of BAPs with internal flow at different oxygenation conditions: (a) Schematic of the experimental setup: MIN6 constructs with internal flow with varying cell density are irrigated with two hollow channels and cultured under perfusion for 2 days under different oxygen tensions. (b) Viability stains of artificial tissue cross sections for one of the channels at varying cell density and oxygen tension. Going from left to right: 10 × 10^6^ cells/mL and 140 mmHg, 40 × 10^6^ cells/mL and 140 mmHg, 40 × 10^6^ cells/mL and 40 mmHg. Live cells are stained green (calcein-AM) and dead cells in red (propidium iodide). (c) Quantification of the viability radius for each condition. **p < 0.01 and ***p < 0.001. (d) Computational model of each oxygenation condition. Visual models are placed in the same order as in (b). (e) Plot of theoretical oxygen concentration as a function of distance from the perfusion channel generated from the computational model. (f) Indication of the theoretical oxygen concentration corresponding to the measured viability radii in each condition. Error bars represent the standard error of the mean, *n* = 3.

In addition to medium samples, tissue monitoring through fluorescent or luminescent reporters would help track tissue viability and functional marker expression in real-time. To demonstrate the adaptability of the system for real-time tissue monitoring through fluorescent reporters, we placed the flow device holding an irrigated artificial pancreatic tissue populated with GFP-expressing MIN6 cells within a small animal imaging instrument (Figure 4(a)). Periodic images taken over 2 days show a gradual decrease in GFP signal, especially in areas further away from the single perfusion channel (Figure 4(b)). Conversely, in the area around the perfusion channel, the signal is maintained throughout the culturing period indicating the emergence of a viability radius. Of note, images taken within the final 12 h appear similar which may indicate a pseudo-steady state during which MIN6 hypoxic cell death no longer occurs and cell expansion is not yet significant, as further substantiated by the quantified fluorescent signal plateau at the 30-h mark (Figure 4(d)). This quantification of cell death kinetics is consistent with our viability (Figures 3(b) and 5) and metabolic analyses (Figure 3(c) and (d)). A viability radius of 0.98 ± 0.06 mm was also observed after sectioning the tissue construct and staining for dead cells (Figure 4(c)).This viability radius is greater than what we report in Table 4 for the same conditions, potentially due to a reduction in oxygen consumption rate (OCR) for the MIN6 reporter line used in these experiments as compared to the unmodified MIN6 cells used in Figure 3(b). Overall, these experiments demonstrate that fluorescent and potentially bioluminescent signals in tissue constructs, can be quantified in real-time at depths of at least 5 mm using our perfusion system.

**Table 4.** Theoretical oxygen concentration corresponding to viability radii observed in artificial pancreatic tissue at different oxygenation conditions.

Oxygen tension (mmHg)	Cell density (10^6^ cells/mL)	Viability radius (mm)	Theoretical oxygen tension at the viability radius (mmHg)
140	10	1.04 ± 0.06	0.93
140	40	0.55 ± 0.02	1.11
40	40	0.28 ± 0.01	1.44

#### Oxygen transport modeling

To explore how the perfusion system could be used to optimize oxygenation and transplantation strategies, we studied how parameters which impact oxygen gradients such as oxygen supply and consumption impact the viability radius. Changing the MIN6 cell seeding density can serve as a model of grafts with different OCR or different device loading, while changing the oxygen tension within the perfused medium can mimic different transplantation sites or anastomosis strategies. As expected, the viability radius significantly increased with lower cell seeding density (*p* = 0.0015 when comparing 40 × 10^6^ cells/mL vs 10 × 10^6^ cells/mL, 140 mmHg) or lower oxygen tension (*p* = 0.0004 when comparing 140 mmHg to 40 mmHg O_2_, 40 × 10^6^ cells/mL) supplied to the perfused medium. To relate these findings to a theoretical model, a finite element model of oxygen mass transport was developed (Figure 5(d) and Table 4). With the model input parameters estimated from past measurements of MIN6 cell oxygen consumption rates and oxygen diffusivity in alginate, a critical oxygen tension related to the value observed at the limit of the viability radius can be estimated to be 1 mmHg (Figure 5(f)).

### Case study II: viability, function, and vascularization in scaffolds

**Figure 6.**
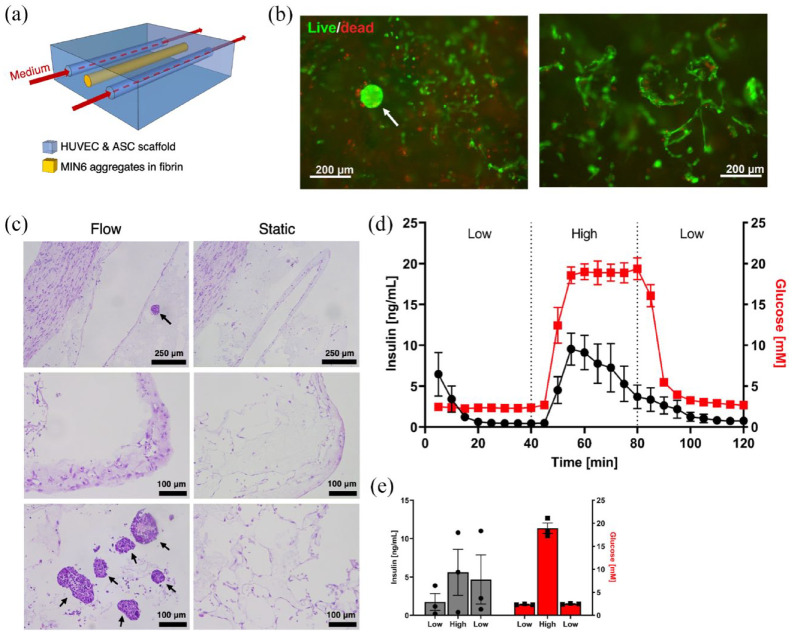
Scaffold with insulin-producing cells within the flow device: (a) Schematic of two polycaprolactone scaffolds were placed in the tissue holder of the flow chamber system. These porous scaffolds contained endothelial cells and adipose-derived stromal cells. In addition, two channels were incorporated for medium flow and one channel for the MIN6 insulin-producing aggregates (0.25 × 10^6^ aggregates/mL). (b) Live/dead staining after 3 days of perfusion culture. Left panel: sectioned scaffold showing a MIN6 aggregate (white arrow) and surrounding HUVEC cells. Right panel: HUVECs at the surface of a scaffold. (c) H&E staining of scaffolds. Top row: scaffold cores. Middle row: scaffold edges. Bottom row: scaffold cores. (d and e) Glucose-stimulated insulin secretion test conducted after 3 days of culture: (d) dynamic and (e) static. The mean and standard error of the mean are plotted, *n* = 3.

A prominent oxygenation strategy for BAPs involves physiological vascularization via vascular ingrowth and the co-transplantation of blood-vessel forming cells or vascular fragments.^72,73^ For our second case study, we use our perfusion system to culture BAPs fabricated using vasculogenic scaffolds. After pre-seeding scaffolds with endothelial cells (HUVEC), MIN6 aggregates suspended in fibrin were seeded into a central cell loading channel. On either side of the central channel with MIN6 and fibrin, two flow-through channels were created to mimic a situation where scaffolds would be vascularized prior to islet delivery. After 3 days of culture, the MIN6 clusters remained highly viable within the scaffolds, both in the flow chamber and the static system, as shown by the live/dead staining (Figure 6(b)). HUVEC cell viability appeared lower within the core of the scaffolds (Figure 6(b)) but highly viable near the surface. After 3 days of culture, HUVECs formed tube-like structures both at the surface and within the core of the scaffolds (Figure 6(b) and (c)). The nuclear staining and extracellular matrix deposition appeared to be higher at scaffold edges under flow as compared to static controls.

Both the perfused and static conditions showed rapid response to high glucose stimulation. However, upon decreasing the glucose concentration, the insulin concentration measured in static controls did not decrease significantly (Figure 6(e)) contrary to the perfused tissues which rapidly showed a reduction in insulin concentration. In addition, the flow chamber provides the ability to carefully monitor the insulin response over time, providing more insight into function without the

### Case study III: viability, function, and metabolite secretion of bioprinted fibers

**Figure 7.**
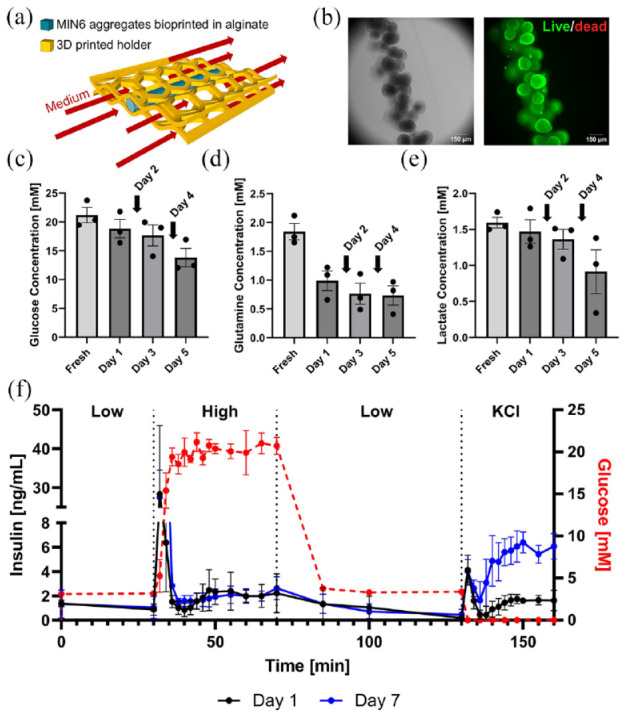
Viability and function of 3D bioprinted pancreatic tissue constructs: (a) Schematic of culturing constructs under flow. Bioprinted fibers are held within the flow field using a custom cage fitted within the tissue holder. (b) Viability stain of MIN6 aggregates after 7 days of culture within the perfusion system. (c–e) Quantification of metabolites in medium samples that were periodically taken after 1, 3, and 5 days of culture under flow. Arrows indicate complete reservoir medium changes (days 2 and 4). Glucose, glutamine, and lactate concentrations are shown, respectively. (f) Dynamic GSIS function of MIN6 aggregates after 1 and 7 days of culture within the perfusion system (300 single MIN6 cells per aggregates; 88,000 aggregates/mL alginate). The mean and standard error of the mean are plotted, *n* = 3.

To apply the perfusion system to devices without internal flow, a custom tissue holder was developed to insert bioprinted fibers into the perfusion system (Figure 7(a)). Fibers with MIN6 cell clusters were maintained in culture for up to 7 days without significant losses in viability (Figure 7(b)). An important increase in insulin release was rapidly observed after 2 min of high glucose stimulation followed immediately by a drop of insulin secretion (Figure 7(f)). A second insulin secretion phase, less important, was observed 10 min after the start of high glucose stimulation and was maintained during all the high glucose stimulation period. A similar pattern was observed during the KCl stimulation with an immediate spike of insulin release before a drop then a second, more constant, phase of secretion. It is to note that the second phase of insulin release after KCl stimulation was higher after 7 days of culture compared to only 1 day, which may reflect MIN6 proliferation or increased insulin content due to hydrogel-induced maturation, as observed previously.^70^ The rapid response times for fibers as compared to devices with internal flow can be explained by the higher surface area/volume ratio. We observed an initial decrease in glucose and glutamine, likely due to their diffusion into the alginate fibers. However, there were no significant differences in glucose, glutamine, and lactate levels in the medium after 1, 3, and 5 days. This is likely because the cells were in excellent condition, as confirmed by viability data, with almost no cell death observed (Figure 7(c) and (d)).

Real-time dynamic GSIS was performed in a similar way with human islets encapsulated in fibers after 2 days of culture and comparing two different oxygen tension (21% and 5%, *n* = 1; Supplemental Figure S2A). Both conditions led to high islet viability (Supplemental Figure S2B), as well as to glucose and KCl-mediated insulin secretion. These preliminary results suggest that basal insulin secretion levels may be lower at 5% O_2_ as compared with 21% O_2_, although experimental replicates would be needed to confirm this observation.

## Discussion

Cell therapy is a promising avenue for reversing T1D with many groups having demonstrated blood glucose normalization after the transplantation of islets or islet-like cells in diabetic rodents and other small animal models.^24,74–77^ However, there is a trend of BAPs being met with limited success when transitioning to humans and larger animals.^17,78^ These results have shed light on the need to better account for the transport of critical compounds such as oxygen and insulin to and from BAPs when working with thicker tissues and higher cell densities.^79^ Upscaling rodent devices to humans can entail changes in cell dosing, device placement, and inflammatory as well as fibrotic responses which can require optimization of oxygenation, device sizing, and other design considerations. All these adaptations can be facilitated with the assistance of computational models along with a robust in vitro perfusion system suitable to different device configurations that approach human tissue length scales.

We have engineered an accessible and highly customizable perfusion system that can be used for the study and development of BAPs in vitro. We show that this system can combine experimental and computational experiments to study cell viability (Figure 5) and function, simulate physiologic oxygen tensions (Figure 5(b–d) and Supplemental Figure S2A), and evaluate vascularization in BAPs that vary in size (mm- to cm-scale) and oxygenation strategy. Furthermore, the system can be used to measure the rapid GSIS kinetics of BAPs which can be maintained within the system for at least one week while retaining sterility (Figure 7(f)). What is particularly advantageous is the ability to conduct multiple GSIS tests on the same specimen and over several days, without introducing contamination. In our experiments, we observed that our bioprinted BAPs exhibited nearly identical responses after 1 or 7 days of culture (Figure 7(f)). Through an integrated sampling port, we also showed that sampling the perfusion medium periodically is possible, allowing analysis of the secreted molecules (Figure 3(c–e)). Since the amount of ATP produced per glucose molecule in anaerobic conditions is significantly reduced compared to aerobic conditions,^80^ impacting beta cell function, it is crucial to precisely define the metabolic state of any pancreatic constructs. It is to be noted that the presence of multiple cell types may influence metabolite secretion. Indeed, islets co-cultured with endothelial cells for example, have demonstrated improved survival through cell signaling interaction.^81,82^ Designing specific studies using the perfusion device would allow the examination of the direct influence of various cell types (such as endothelial or immune cells) on islet metabolite secretion.

To study the kinetics of cell viability or function in vitro, cells engineered to express fluorescent or bioluminescent signals can replace islets cells within BAP constructs. Using a small animal imaging chamber, we measured the time-dependent effects of oxygenation on cell viability in perfusable tissue constructs were quantified via time-lapse fluorescence imaging (Figure 4). One important limitation of the real-time viability is the requirement for a fluorescent cell line, meaning it can be specifically used for research tests in vitro or in vivo at the preclinical level. This work highlights the capacity of our perfusion system to facilitate real time measurements that could be used to monitor other cell types and other tissue engineering-related processes such as differentiation, cell secretive function,^83,84^ and molecule diffusion^85^ within BAPs.

This work is part of the growing body of evidence that oxygenation is critical for BAP function when transitioning into the human scale. Reliable oxygenation is essential for BAP survival and function but can be influenced by a number of variables.^13^ The supply of oxygen to BAPs hinges on oxygenation strategies and transplantation location whereas the consumption of oxygen depends on the cell density and cell type. The biomaterials used for BAP fabrication also influence the rate at which oxygen travels from the source to the cells. Using both analytical and computational models, the limitations of oxygen transfer within BAPs have been outlined by other groups^48,86–88^ and suggest that these dynamics dictate the limitations of BAP geometry and size. Although these findings have been impactful, a route to easily validate models of BAP oxygenation and integrate them experimentally has been missing in the literature. Coupling computational models with in vitro testing can accelerate the development of successful human-scale devices and better prepare BAPs for clinical trials while decreasing the reliance on small animal testing.

We demonstrate that our perfusion system can be used to experimentally evaluate different oxygenation parameters for BAP testing, including cell density and oxygen partial pressure supplied via internal flow channels (Figure 5). We observed a remarkably clear viability radius that can be modulated via these parameters and compared to numerical oxygen transport models. This approach defined an estimate of the oxygen partial pressure cutoff value for MIN6 survival of ~1 mmHg (Table 4) which is consistent with the literature.^89,90^ This approach could be applied to identify the cutoff value for other insulin-producing cell sources. The viability radius was remarkably consistent across experimental conditions, with real-time fluorescence imaging (Figure 4) as well as nutrient consumption rates (Figure 3(c–e)) suggesting that a steady state (i.e. the final viability radius) is reached after ~30 h for MIN6 cells. In addition to viability, we also showed that culturing islets at low oxygen tension (38 mmHg) reduced the amount of insulin secreted in response to glucose compared to the normoxic culture (140 mmHg) in Supplemental Figure S2A.

**Figure 8.**
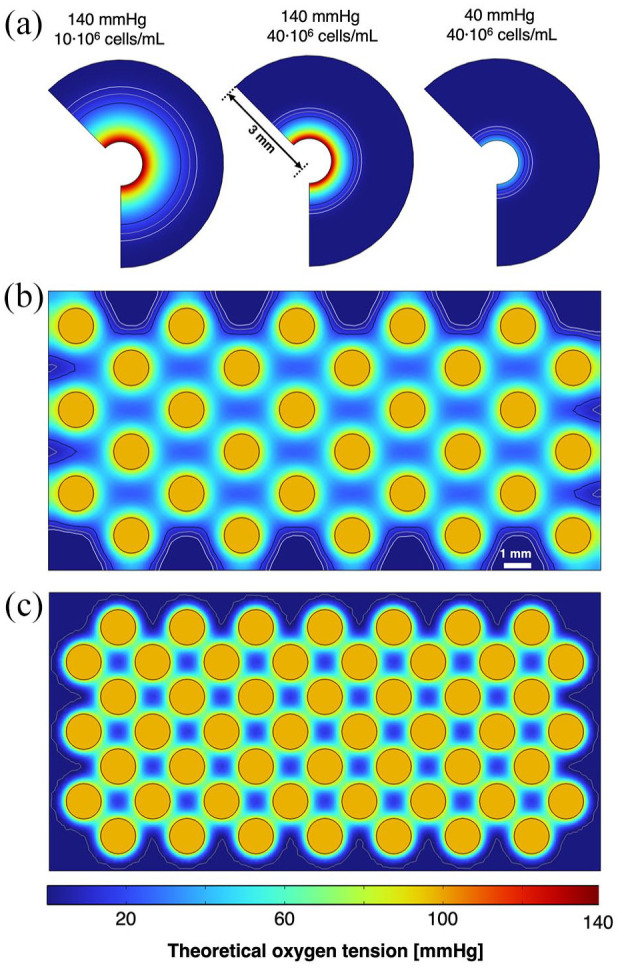
Analysis of the theoretical viability radius in perfusable BAPs containing islet cells and MIN6: (a) Computational model of theoretical cross sections for BAPs containing islet cells at varying cell density and oxygen tension. Going from left to right: 10 × 10^6^ cells/mL and 140 mmHg, 40 × 10^6^ cells/mL and 140 mmHg, 40 × 10^6^ cells/mL and 40 mmHg. (b and c) Theoretical oxygen tension of perfusable BAPs (40 × 10^6^ cells/mL) within the largest cross section possible in our current perfusion system. The oxygen tension within the perfusate was set to arterial levels (100 mmHg). Channels are arranged in a simple array by way of maximizing the viable area. (b) With human islet cells: black, brown, and white contours indicate oxygen tensions of 20, 10, and 5 mmHg which have been reported as the minimal oxygen tension required for islet cell survival. (c) With MIN6 cells: the contour line indicates an oxygen tension of 1 mmHg.

Although the MIN6 cell line is a valuable tool to determine insulin kinetics and cell viability in vitro, they become less relevant in clinical settings. In particular, the OCR of MIN6 cells has been reported to be approximately four times greater than primary human islets^40^ and be more sensitive to hypoxia. Simulating the oxygen tension of islets cells within the perfusable BAPs presented earlier (Figure 8(a)) have suggested devices with islet cells may demonstrate larger viability radii (Table 5) than those measured for MIN6 cells (Table 3) despite the greater sensitivity to hypoxia. Moreover, when simulating the perfusable network required to oxygenate most of a 2 cm x 1 cm rectangular slab (i.e. the maximum frontal cross section within our current flow device), channels could be spaced out further in BAPs containing islets (Figure 8(b)) and fewer channels were needed when compared to the MIN6 BAP (Figure 8(c)). This suggests that the lower OCR in human islets plays a more influential role in determining the viable regions in BAPs than the lower tolerance for hypoxia in perfusable BAPs when compared to MIN6 cells. A caveat of these findings is that although islets may require oxygen tensions of up to 20 mmHg to survive,^91–93^ greater oxygen tensions have been reported for the proper function of islets. As such, to generate a functional BAP containing human islets, a denser network of channels would be required. This analysis substantiates the claims within the tissue engineering field that complex networks akin to physiological vasculature would be required to maintain thick, cm-scale tissues suitable for human applications.^94–96^

**Table 5.** Theoretical viability radius of human islet cells within perfusable BAPs.

Oxygen tension cutoff (mmHg)	Theoretical viability (mm)		
	140 mmHg	140 mmHg	40 mmHg
	10 × 10^6^ cells/mL	40 × 10^6^ cells/mL	40 × 10^6^ cells/mL
5	1.575	0.875	0.375
10	1.375	0.765	0.315
20	1.125	0.625	0.155

A major advantage of the perfusion device is its flexibility in culturing BAP constructs with different oxygenation strategies, scales, and geometries. Devices with internal flow facilitate active oxygen transport through blood flow which is an attractive strategy for maintaining cell survival at high density.^20,54^ To promote the development of BAPs with internal flow, our perfusion system can subject constructs to physiological oxygen tensions and establish a pressure gradient across tissues to drive laminar flow similar to that observed in blood vessels (Figure 2). Microvascularized BAPs incorporate blood vessels through angiogenesis and/or vasculogenesis to ensure sufficient oxygen supply.^16^ An example of this approach is the incorporation of microvessels fragments within BAPs which has been shown as an effective strategy to quickly grow microvessel networks that are connected to the host vasculature within constructs and reverse diabetes in immunocompetent mice.^22^ In our case study II, the 3 days co-culture of islets and endothelial cells is insufficient to form mature blood vessels and ensure adequate interactions between the two cell types. As reported by others, mature blood vessels typically require at least 7 days to develop in vitro or in vivo using microvessel co-transplantation techniques.^22,95^ As shown in Figure 7, we are able to maintain cultures for a 7-day period, which aligns with the requirements for a vascularized islet encapsulation strategy. The capacity to culture BAPs for at least 7 days and the option to monitor tissues in real-time using the imaging window in our perfusion system could be a valuable tool to study blood vessel formation kinetics within microvascularized BAPs. Our system’s capacity to study angiogenesis and vasculogenesis within artificial tissue constructs opens the possibility to explore which cell types are involved in the vascularization of artificial tissues and how different cells interact with each other, as well as with different materials/geometries. While BAPs without internal flow depend on diffusive oxygen transport, they also facilitate flexibility in possible transplantation sites. Leveraging this flexibility can offer distinct advantages in minimizing immune responses and promoting safety and ease of retrieval/monitoring.^97^ The oxygen tension of implantation sites significantly affects the thickness at which cells can remain functional within BAPs thereby impacting the size, cell density, and geometry of these devices. Considering that oxygen tensions can vary between implantation sites, our perfusion system can streamline the development of BAPs without internal flow by enabling long term culture and evaluation of GSIS function at oxygen tensions reflective of different implantation sites.

Indeed, the experiments presented in this article demonstrate a proof of concept that our perfusion system can be used to culture tissue constructs for 2–7 days. However, it has been highlighted that physiological processes such as stem cell-derived beta cell maturation,^9,98^ vascularization, and anastomosis^99^ can take weeks and even months. As such, longer culture times within the perfusion system could facilitate more reliable evaluations of tissue construct function and provide insights of how implants may mature in the long term. One consideration when preparing for longer culture times is the maintenance of sterility. To address this, a robust sterilization procedure should be developed, and reliable aseptic techniques should be adopted especially, when opening the closed system for exchanging medium in the reservoir and collecting samples for the loop. Another possible obstacle, particularly in studying the viability radius and oxygenation could be the proliferation of cell lines that would disrupt the steady state of the oxygen dynamics. Cell lines with low turnover such as primary beta cells^100^ or genetically engineered cell lines with conditional proliferation^101,102^ can be incorporated into BAPs to minimize dynamic behaviors in oxygen consumption and overall transport. Considering that alginate lacks the capacity to interact with cells^103^ in a manner that extracellular matrix (ECM) does in situ, longer culture times for our constructs could be limited by an inability provide a suitable cellular microenvironment. As such, alginate composites such as those consisting of RGD peptides and collagen^104^ may be required to promote cell longevity and long-term function. Of note, alginate composites like those including pectin can improve the immunoprotective properties of tissue constructs in addition to in vivo biocompatibility.^105^ Of note, we demonstrated in Figures 3 and 7 that metabolite concentrations can be dynamic and molecules such as lactate from glycolysis can build up in the system that could result in an inconsistent culturing environment. In future versions of our system, an inlet flow and bleed line could be introduced into the loop as a means of siphoning off a build-up of metabolites and creating a more constant condition. Beyond BAP engineering, our perfusion system could serve as a valuable tool for the development of other bioartificial organs, the study of oxygenation on stem cell differentiation, and even for drug discovery applications.^106–110^

## Conclusion

This work aims to address the challenges related to transitioning BAPs from lab to clinical scale. The disconnection between the promising results of BAPs in small animal models and the limited success in humans has emphasized the need for more robust preclinical testing and tissue engineering methodologies. Namely, limitations regarding insulin delivery and oxygenation are prominent in BAPs likely due to the changes in surface area to volume ratio when transitioning to human scale. As such, we present a perfusion system capable of culturing cm-scale BAPs, real-time monitoring, and GSIS function testing. Our system also accommodates a range of tissue oxygenation strategies and can mimic physiological oxygen tensions. While in vitro settings will never fully replicate the in vivo environment, history has shown that the refinement of BAPs has been a missing piece for a successful transition from rodent models to humans.

## Supplemental Material

sj-docx-1-tej-10.1177_20417314241284826 – Supplemental material for Oxygenation and function of endocrine bioartificial pancreatic tissue constructs under flow for preclinical optimization Supplemental material, sj-docx-1-tej-10.1177_20417314241284826 for Oxygenation and function of endocrine bioartificial pancreatic tissue constructs under flow for preclinical optimization by Brenden N Moeun, Florent Lemaire, Alexandra M Smink, Hamid Ebrahimi Orimi, Richard L Leask, Paul de Vos and Corinne A Hoesli in Journal of Tissue Engineering
