# Supplementary material for: Oxygenation and function of endocrine bioartificial pancreatic tissue constructs under flow for preclinical optimization
Source: J Tissue Eng. 2025 Jan 23;16:20417314241284826. doi: 10.1177/20417314241284826 (PMC11758540; doi:10.1177/20417314241284826)
Supplement: sj-docx-1-tej-10.1177_20417314241284826 – Supplemental material for Oxygenation and function of endocrine bioartificial pancreatic tissue constructs under flow for preclinical optimization [file sj-docx-1-tej-10.1177_20417314241284826.docx]

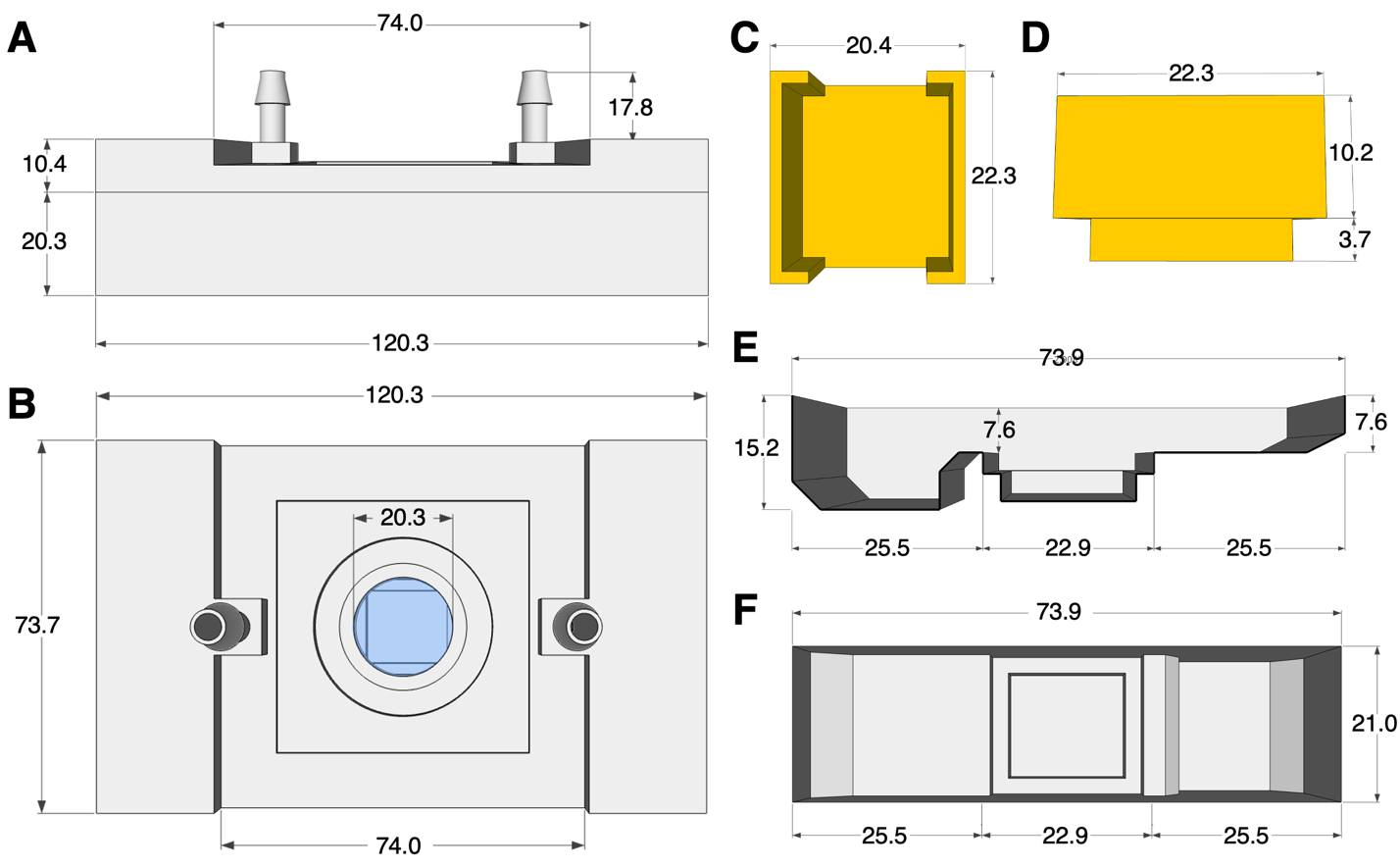


**Figure S1.** Dimensions of flow device and components. **(A)** longitudinal view of assembled flow device, **(B)** top view of assembled flow device, **(C)** top view of tissue holder, **(D)** longitudinal view of tissue holder, **(E)** longitudinal cross -section of internal flow chamber, and **(F)** top view of the internal flow chamber.


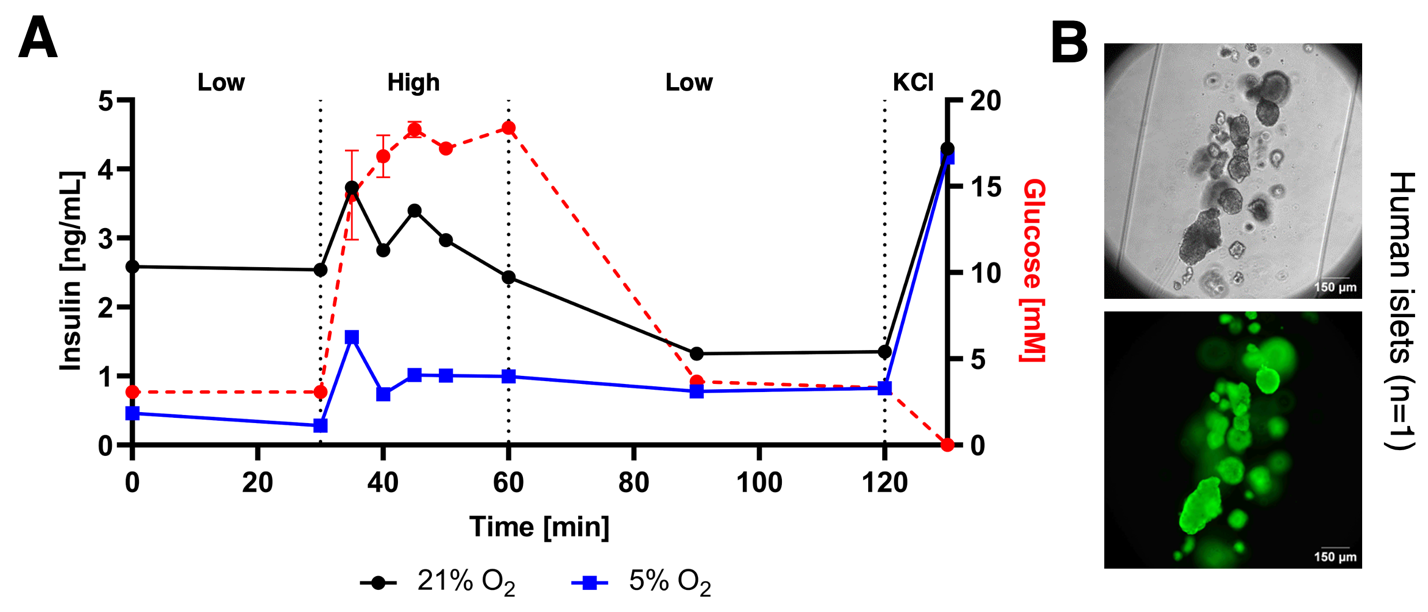


**Figure S2.** Viability and function of 3D bioprinted human islet constructs:**(A)** Dynamic GSIS function human primary islets at atmospheric and venous oxygen tensions (44 000 human islets/ml alginate), n=1; **(B)** Viability stain of primary human islets after 2 days of culture within the perfusion system.
